# Whole-Exome Sequencing to Decipher the Genetic Heterogeneity of Hearing Loss in a Chinese Family with Deaf by Deaf Mating

**DOI:** 10.1371/journal.pone.0109178

**Published:** 2014-10-07

**Authors:** Jie Qing, Denise Yan, Yuan Zhou, Qiong Liu, Weijing Wu, Zian Xiao, Yuyuan Liu, Jia Liu, Lilin Du, Dinghua Xie, Xue Zhong Liu

**Affiliations:** 1 Department of Otolaryngology-Head and Neck Surgery, Institute of Otology, the Second Xiangya Hospital, Central South University, Changsha, Hunan, China; 2 Departments of Otolaryngology-Head and Neck Surgery, Leonard M. Miller School of Medicine, University of Miami, Miami, Florida, United States of America; Innsbruck Medical University, Austria

## Abstract

Inherited deafness has been shown to have high genetic heterogeneity. For many decades, linkage analysis and candidate gene approaches have been the main tools to elucidate the genetics of hearing loss. However, this associated study design is costly, time-consuming, and unsuitable for small families. This is mainly due to the inadequate numbers of available affected individuals, locus heterogeneity, and assortative mating. Exome sequencing has now become technically feasible and a cost-effective method for detection of disease variants underlying Mendelian disorders due to the recent advances in next-generation sequencing (NGS) technologies. In the present study, we have combined both the Deafness Gene Mutation Detection Array and exome sequencing to identify deafness causative variants in a large Chinese composite family with deaf by deaf mating. The simultaneous screening of the 9 common deafness mutations using the allele-specific PCR based universal array, resulted in the identification of the 1555A>G in the *mitochondrial DNA (mtDNA) 12S rRNA* in affected individuals in one branch of the family. We then subjected the mutation-negative cases to exome sequencing and identified novel causative variants in the *MYH14* and *WFS1* genes. This report confirms the effective use of a NGS technique to detect pathogenic mutations in affected individuals who were not candidates for classical genetic studies.

## Introduction

Hereditary hearing loss is highly heterogeneous, with hundreds of mutations in more than 60 genes found to disrupt auditory function (http://hereditaryhearingloss.org). Yet it is estimated that for the majority of patients with a presumed genetic deafness the etiology is still undetermined. In many of those unsolved cases, the cause may involve a known or a novel mutation in currently known causative genes. However, because of practical limitations including the large number and size of many genes and the high cost and cumbersomeness of Sanger sequencing, these genes are not routinely screened in a diagnosis setting. Although for many decades, genome wide linkage analysis has been used for disease gene identification, this approach is time consuming and unsuitable for small families with an inadequate number of affected individuals. Moreover, other traditional strategies including candidate gene study, combined linkage study and positional cloning have been very challenging in detecting extremely rare cases or sporadic cases caused by *de novo* mutation. As a result, the causal variant for these hearing-impaired patients has remained elusive and the genes for many human deafness associated loci have not been identified.

Recent developments in high-throughput sequence capture methods and next-generation sequencing (NGS) technologies have now made Whole exome sequencing (WES) technically feasible and more cost-effective to elucidate the genetic basis of Mendelian disorders with hitherto unknown etiology and diseases with genetic and phenotypic heterogeneity [1]. Targeted or WES has proved to be a robust and powerful tool to discover mutations or genes for both nonsyndromic and complex syndromic forms of hearing loss, especially in small families with a distinct and particular phenotypic expression that were once too small to map [2]–[4].

A typical WES screen will identify between 20,000 and 50,000 exonic variants [5]. A multitude of programs and databases are thus required to handle such a wealth of information. NGS data analysis normally involves applying multiple filters to the data in order to prioritize candidate functional variants from the large pool of candidates. With regard to deafness, because of its extreme genetic heterogeneity, WES analysis of several affected and healthy relatives from one family may be powerful strategy to discover rare causative genes or mutations. In the present study, we applied WES to determine the deafness causative genes and mutations in a large composite Chinese family with assortative mating. Among the mutations detected, we identified the 1555A>G mutation in the *mtDNA 12S rRNA* gene and novel mutations (c.541G>A; p.A181 T and c.449C>T; p.A150 V) in the genes encoding MYH14 and WFS1, respectively.

## Materials and Methods

### Clinical Evaluation

A large Chinese family with nonsyndromic sensorineural hearing loss was identified through the Department of Otolaryngology-Head and Neck Surgery, the Second Xiangya Hospital of Central South University, China. All patients underwent a complete history and physical exam as well as audiogram including air and bone (AC/BC) conduction pure tone thresholds, auditory brainstem response (ABR) thresholds, and auditory steady state response (ASSR). Audiological assessment was performed by measuring the average hearing thresholds level at 500, 1000, and 2000 Hz. High-resolution, thin-section computed tomography (CT) and magnetic resonance imaging (MRI) of the temporal bone were used to detect any congenital malformations in two children (IIIC:2 and IIIC:3) prior to cochlear implant surgery. This study was approved by the Ethics Review Committee of the Second Xiangya Hospital of Central South University. And the written consent form of the study was obtained from the study participants or their guardians.

### Preparation of DNA

Peripheral blood samples were collected from patients and their lineal relatives. Genomic DNA was then extracted from the blood using RelaxGene Blood DNA System (TIAGEN Biotech, Beijing, China).

### Simultaneous deafness gene mutation detection using microarray

Hereditary hearing loss allele-specific PCR based universal array (ASPUA) (CapitalBio, Beijing, China) was used to simultaneously screen 9 mutations causing hereditary hearing loss (*GJB2*: c.35delG, c.176del16, c.235delC, c.299-300delAT; *GJB3*: c.538C>T (R180X); *SLC26A4*: c.IVS7-2A>G, c.2168A>G (p.H723R); *mtDNA 12S rRNA*: 1555A>G, 1494C>T). Multiplex allele-specific PCR was performed and chips were imaged with a LuxScan TMHT 24 Microarray Scanner (CapitalBio, Beijing, China).

### Whole exome sequencing and data analysis

Enrichment of coding exons and flanking intronic regions was performed using a solution hybrid selection method with the SureSelect human all exon 50 Mb kit (Agilent Technologies) following the manufacturer’s standard protocol. Adapter sequences for the Illumina Hiseq2000 were ligated and the enriched DNA samples were sequenced on the Hisq2000 instrument (Illumina). The resulting data were processed and annotated with Burrows-Wheeler Aligner (BWA), Genome Analysis Toolkit (GATK), and SeattleSeq. Details of read mapping, variant detection, filtering and annotation have previously been described [6].

### *GJB2, TRMU* genes analysis and candidate variants validation by direct Sanger sequencing

Whole coding region of the *GJB2* gene and the exon 1 of the *TRMU* gene were analyzed using previously reported primers [7]. Positive sequence variants detected in WES were tested for segregation in the family by first performing Sanger sequencing of the DNA of affected members. If all affected members were found to be carriers of the sequence variant, unaffected members of the family were also sequenced.

The primers were designed using Primer3, v.0.4.0 (http://frodo.wi.mit.edu/). Polymerase Chain Reaction (PCR) reactions included 10–40 ng of genomic DNA with Taq DNA polymerase (Roche), were performed using standard methods, and sequenced using the ABI PRISM Big Dye Terminator Cycle Sequencing V3.1 Ready Reaction Kit and the ABI PRISM 3730 DNA Analyzer (Applied Biosystems).

## Results

### Clinical manifestation

**Figure 1:**
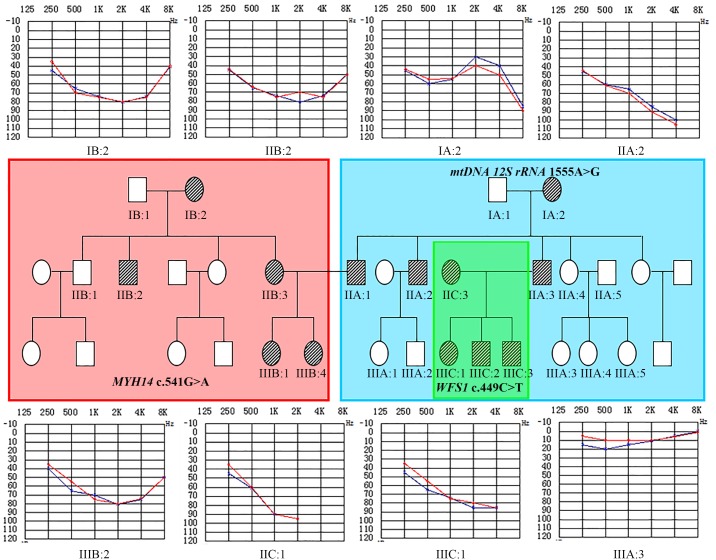
Pedigrees of the composite family and Audiograms. Segregation of the *mtDNA 12S rRNA* 1555A>G mutation (A), c.541G>A; p.A181 T in *MYH14* (B) and c.449C>T; p.A150 V in *WSF1* (C). Individuals for whom DNA samples are available and tested are labeled with their identification number. Audiograms of eight individuals exhibiting bilateral, symmetric, sensorineural hearing loss are shown. The blue curves indicate the left ear and red curves indicate the right ear.

Genealogy of the composite family with deaf individuals is shown in Fig. 1. Physical examination of all members did not demonstrate any dysmorphic features suggestive of syndromic disease. A computed tomography (CT) scan and magnetic resonance tomography (MRI) performed in subjects IIIC:2 and IIIC:3 prior to cochlear implant surgery and did not show any abnormalities. Age at onset of hearing impairment in affected individuals varied from congenital to fourth decades of life. They exhibited symmetric progressive sensorineural hearing loss that ranged from moderate to profound in degree with sloping, flat or U-shaped audio profiles. One affected male (IIA:3) had a history of aminoglycoside exposure (Table 1; Fig. 1).

**Table 1:** The clinical features and the genetic study of the composite Chinese family with assortative mating.

Number	Sex	Age at onset	Hearing test (BC^1^/ASSR^2^)	Audiogram profiles	*mtDNA 12S rRNA* 1555A>G	*MYH14*		*WFS1* c.449C>T (p.A150 V)
						c.541G>A (p.A181 T)	c.2594C>T (p.T865 M)	
IA:1	Male	-	L^3^∶20 dB; R^4^∶25 dB	normal	wt	wt^5^/wt	wt/wt	wt/wt
IA:2	Female	The fourth decade	L:51 dB; R:55 dB	sloping	Homoplasmic	wt/wt	wt/wt	wt/wt
IIA:1	Male	The first decade	L:>100 dB; R:>100 dB	sloping	Homoplasmic	wt/wt	wt/wt	wt/wt
IIA:2	Male	The fourth decade	L:70 dB; R:70 dB	sloping	Homoplasmic	wt/wt	wt/wt	wt/wt
IIIA:1	Female	-	L:20 dB; R:20 dB	normal	wt	wt/wt	wt/wt	wt/wt
IIIA:2	Male	-	L:20 dB; R:23 dB	normal	wt	wt/wt	wt/wt	wt/wt
IIA:3	Male	The first decade	L:90 dB; R:98 dB	sloping	Homoplasmic	wt/wt	wt/wt	wt/wt
IIA:4	Female	-	L:25 dB; R:20 dB	normal	Homoplasmic	wt/wt	wt/wt	wt/wt
IIA:5	Male	-	L:20 dB; R:20 dB	normal	wt	wt/wt	wt/wt	wt/wt
IIIA:3	Female	-	L:23 dB; R:20 dB	normal	Homoplasmic	wt/wt	wt/wt	wt/wt
IIIA:4	Female	-	L:25 dB; R:25 dB	normal	Homoplasmic	wt/wt	wt/wt	wt/wt
IIIA:5	Female	-	L:23 dB; R:20 dB	normal	Homoplasmic	wt/wt	wt/wt	wt/wt
IB:1	Male	-	L:25 dB; R:23 dB	normal	wt	wt/wt	wt/wt	wt/wt
IB:2	Female	The first decade	L:70 dB; R:75 dB	U-shape	wt	mt^6^/wt	wt/wt	wt/wt
IIB:1	Male		L:20 dB; R:20 dB	normal	wt	wt/wt	wt/wt	wt/wt
IIB:2	Male	The first decade	L:85 dB; R:81 dB	U-shape	wt	mt/wt	wt/wt	wt/wt
IIB:3	Female	The first decade	L:85 dB; R:80 dB	U-shape	wt	mt/wt	wt/wt	wt/wt
IIIB:1	Female	The first decade	L:83 dB; R:>100 dB	U-shape	wt	mt/wt	mt/wt	wt/wt
IIIB:2	Female	The first decade	L:80 dB; R:80 dB	U-shape	wt	mt/wt	mt/wt	wt/wt
IIC:1	Female	congenital	L:90 dB; R:90 dB	sloping	wt	wt/wt	wt/wt	mt/wt
IIIC:1	Female	congenital	L:90 dB; R:85 dB	sloping	wt	wt/wt	wt/wt	mt/wt
IIIC:2	Male	congenital	L:>100 dB; R:>100 dB	-	wt	wt/wt	wt/wt	mt/wt
IIIC:3	Male	congenital	L:>100 dB; R:>100 dB	-	wt	wt/wt	wt/wt	mt/wt

1 Bone conduction;

2 Auditory steady state response;

3 left ear;

4 Right ear;

5 wild type;

6 mutant allele.

### Allele-specific PCR-based universal array for simultaneous deafness associated genes detection

**Figure 2:**
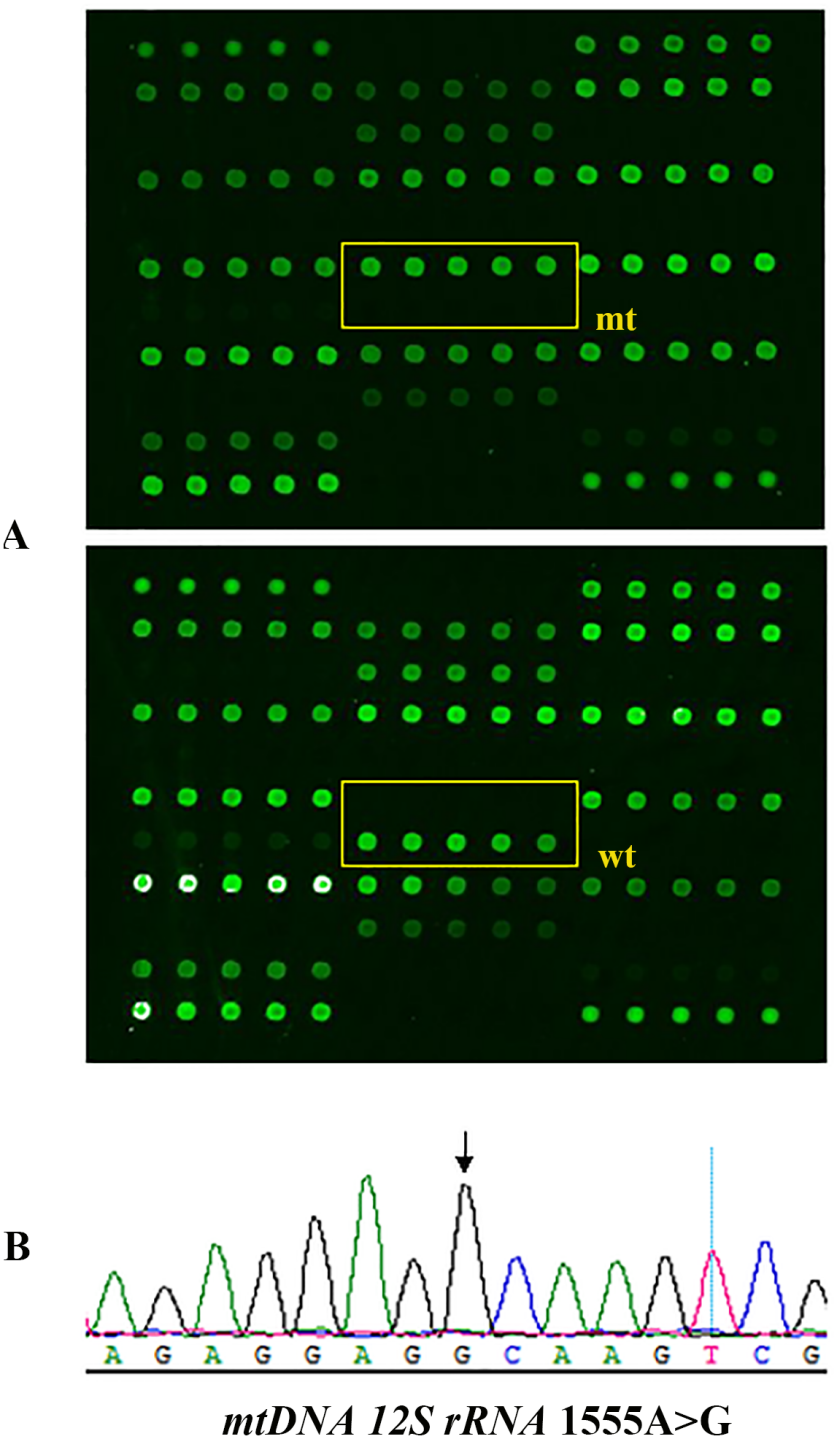
Deafness genes detection array. The *mtDNA 12S rRNA* 1555A>G homoplasmic mutation. The box in the scanned image of the microarray chip represents the *mtDNA 12S rRNA* 1555A>G square areas. The upper dark dots indicate the wild-type is absent and the green dots below indicate the 1555A>G homoplasmic mutation (A). Sequence chromatograms showing the homoplasmic 1555A>G mutation as indicated by the arrow (B).

Among the individuals (IIC:1; IIA:3; IIIC:1; IIIC:2; IIIC:3) tested for hotspot mutations, only one affected subject (IIA:3) was found to carry the *mtDNA 12S rRNA* 1555A>G mutation in homoplasmic state (Table 1 and Fig. 2A). Sanger sequencing analysis of the *mtDNA* of other family members revealed the presence of a homoplasmic c.1555A>G mutation in IA:2; IIA:1; IIA:2; IIA:3; IIA:4; IIIA:3; IIIA:4; IIIA:5 (Table 1; Fig. 2B), but the mutation was absent in other affected individuals (IIB:3; IIIB:1; IIIB:2; IIC:1; IIIC:1; IIIC:2; IIIC:3) and family members with normal hearing (IIA:5; IIIA:1; IIIA:2) (Fig. 1B and 1C). In addition, no pathogenic mutations were identified in either the coding region of the *GJB2* gene or the exon 1 of the *TRMU* gene by Sanger sequencing in any of the affected individuals tested. Individuals IIA:4, IIIA:3, IIIA:4 and IIIA:5 have normal hearing despite harboring the 1555A>G homoplasmic mutation. These individuals had no known history of aminoglycoside exposure. It has become clear that mtDNA mutations can cause nonsyndromic hearing loss (NSHL), both as a primary or as a predisposing factor. In the absence of exposure to aminoglycosides, the age of onset associated with the 1555A>G mutation may differ considerably among members of the same family. The clinical phenotype can also be variable, ranging from normal hearing, progressive moderate hearing loss starting in adult life, to profound congenital deafness [8]. It has been suggested that certain nuclear modifiers such as the 35delG mutation in the *GJB2* gene and the A10 S mutation in the *TRMU* gene as well as certain mitochondrial haplogroups may modulate the phenotypic expression of the hearing loss associated with the 1555A>G mutation [8], [9]. In the present study, we have excluded the role of other common variants in the *mtDNA 12SrRNA* gene and *GJB2* gene in the 1555A>G positive individuals using the Deafness Gene Mutation Detection Array and Sanger sequencing. Thus, the observed variable phenotypic expression could be attributed to other modifier genes. IIA:1 and IIA:2 are both deaf and carry the homoplasmic 1555A>G mutation. Hearing loss is more likely caused by aminoglycoside ototoxicity in these cases. So far, the only well documented environmental factor affecting the 1555A>G mutation are the aminoglycosides. This antibiotic class is widely used in developing countries like China, India and South Africa as a first-line treatment against multi-drug resistant tuberculosis due to its broad spectrum activity and cost-effectiveness.

### WES

The DNA samples from 2 distant relatives from the third generation (IIIB:2 and IIIC:2) were used for identification of the causative mutations by WES. Approximately, 94,000 single nucleotide variants and 10,206 INDELS per sample were obtained before variant filtering. Coverage of targeted exons for >10 reads were ranged from 92.3% to 93.5% and >20 reads from 81% to 83.5%. The Genomes Management Application (GEM.app), University of Miami Miller School of Medicine (https://secureforms.med.miami.edu/hihg/gem-app) was applied for data filtering. After analysis and filtering of the data according to the dominant inheritance model, 48 and 35 non-synonymous variants in 21 known deafness genes were detected in affected subject (IIIB:2) and (IIIC:2), respectively. The numbers of variants were further narrowed down by excluding the variants reported in dbSNP137, NHLBI (http://evs.gs.washington.edu/EVS/) or that had a minor allele frequency of more than 0.05% in these databases. Computational functional prediction algorithms (PolyPhen, SIFT, MutationTaster) and conservation scores (PHASTCO, GERP, PHYLOP) were also applied. By this filtering strategy, we have rapidly identified two missense mutations (c.541G>A; p.A181 T and c.2594C>T; p.T865 M) in the *MYH14* gene in IIIB:2 and a missense variant (c.449C>T; p.A150 V) in the *WFS1* gene in IIIC:2.

By filtering the variants according to the inheritance model (autosomal recessive with both homozygous and compound heterozygous), only a homozygous mutation (c.301G>A) in the *C3orf17* gene was found in affected subject IIIC:2. This gene is located on 3q13.2 and code for an uncharacterized single**-**pass transmembrane protein, consisting of 567 amino acid residues.

### Expanded familial validation and Sanger sequencing confirmation

**Figure 3:**
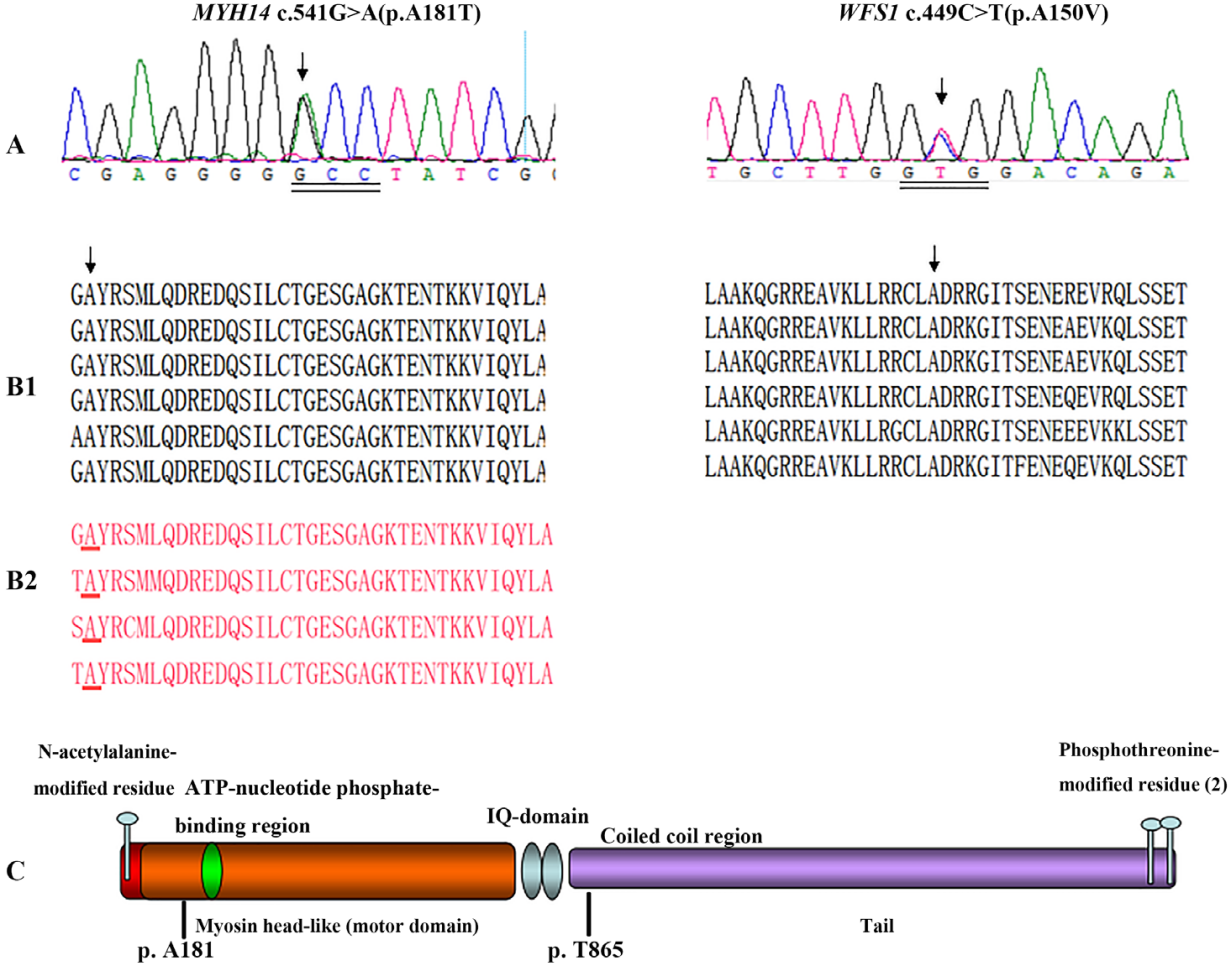
Mutations analysis and conservations of the identified variants in the *MYH14* and *WFS1* genes. Chromatogram of exon 3 of the *MYH14* gene showing heterozygous mutation c.541G>A in affected individuals (left panel; arrow) and heterozygous mutation c.449C>T in exon 4 of the *WFS1* gene (right panel; arrow) (A). Protein sequence alignment showing conservation of residues MYH14 A181 (left panel; arrow) and WFS1 A150 (right panel; arrow) across six species. Sequence alignment of the non-muscle class II myosin showing conservation of MYH14 A181 (left panel; red underlined) (B). Diagram of the human MYH14 consisting of a N-terminal myosin domain, a myosin head region, a motor domain, two IQ motifs, a coiled-coil region and a tail domain (C).

To validate the WES results from probands, we performed Sanger sequencing on extended family members. Expanded familial validation revealed that the sequence variation *MYH14* c.541G>A at codon 181 (exon 3) (Fig. 3A) located in the motor domain, near the ATP binding site of the *MYH14* gene (Fig. 3C), co-segregates with the deafness phenotype in all affected individuals (IB:2, IIB:2, IIB:3, IIIB:1 and IIIB:2) in part B of the composite pedigree (Fig. 1B), while the variant c.2594C>T; p.T865 M presents only in subjects IIIB:1 and IIIB:2. This sequence change is neither present in the exome variant server (EVS) database (6448 exomes; http://evs.gs.washington.edu/EVS/) nor in dbSNP (http://www.ncbi.nlm.nih.gov/projects/SNP/). It was absent in the parents and is thus more likely a *de novo* sequence change (Table 1). A number of studies have shown that a significant fraction of single-nucleotide variations (SNVs) occur *de novo*, representing the most extreme form of rare variants [10]–[12]. One of the superiorities of exome sequencing is the capacity of finding such rare SNVs. Furthermore, Sanger sequencing validated the proband’s (IIIC:2) mutation (c.449C>T; p.A150 V) in the *WFS1* gene and demonstrated that the two siblings (IIIC:1 and IIIC:3) and mother (IIC:1) also carry this mutation (Table 1; Fig. 1C). The amino acids A181 in MYH14 and A150 in WFS1 are conserved in all sequenced vertebrates, from zebrafish to mammals (Fig. 3B1). Furthermore, Sequence alignment of the non-muscle class II myosin showed conservation of MYH14 p.A181 (Fig. 3B2). The mutations were predicted to affect protein function, with polyphen 2 score of 0.99 and SIFT score of 0.01 for MYH14 p.A181 T and polyphen 2 score of 0.964 for WFS1 p.A150 V [13]. Neither of these variants was identified in a panel of 320 normal hearing Chinese control subjects. The (c.541G>A; p.A181 T) in the *MYH14* gene was not present in the exome variant server (EVS) database (6448 exomes) or in the dbSNP (http://www.ncbi.nlm.nih.gov/projects/SNP/). The WFS1 (c.449C>T; p.A150 V) is carried by 1 out of 6489 exomes present on the Exome Variant server (MAF = 0.0077%). These observations suggest that the mutations A181 T in MYH14 and A150 V in WFS1 are likely to have a detrimental effect on the protein and its function. Finally, the variant (c.301G>A; pE101 K) in the *C3orf17* gene detected in both subjects with and without hearing loss was excluded as a deafness causative gene.

## Discussion

The simultaneous screening of target genes of interest allows for an efficient and cost-effective method of analyzing panels of genes concurrently, as opposed to testing on a gene-by-gene basis as occurs in Sanger sequencing. This is especially true for a disorder with heterogenic pathology, such as hearing loss (HL). On a larger scale, WES has become a promising tool for detecting novel and known mutations involved in hereditary deafness and for solving many deafness cases, as it screens the exons of all genes in the human genome. In the present study, we first tested the subjects IIA:3, IIC:1, IIIC:2 and IIIC:3 for deafness common mutations, using a targeted microarray, which resulted in the identification of the 1555A>G mutation in the *mtDNA 12S rRNA* gene in individual IIA:3 in a homoplasmic state. To further assess the mutation-negative cases, we performed exome sequencing with DNA from IIIB:2 and IIIC:2. After applying a filtering strategy using GEM.app followed by validation and segregation analysis of the variants in the family, we identified new deafness causative mutations in the *MYH14* and *WFS1* genes in part B and C of the composite family, respectively (Table 1 and Fig. 1). Defects in these genes have previously been described to cause autosomal dominant hearing loss.

The *MYH14* gene encodes nonmuscle myosin heavy chain II-C (NMHCII-C), a member of the NMHCII family of ATP-dependent molecular motor which interacts with cytoskeletal actin and regulates cytokinesis, cell motility, and cell polarity [14]. The *MYH14* encoded a protein that contains a N-terminal myosin domain, a myosin head-like region, two IQ domains, and a C-terminal myosin tail (Fig. 3C). It shares significant similarity with MYH9, MYH10, and MYH11, with highest conservation in the myosin head domain. The amino acid residue at the position 181 is highly conserved between species (Fig. 3B1) and among members of NMHC II, including MYH9, MYH10 and MYH11 (Fig. 3B2). The replacement of the hydrophobic, non-polar residue Alanine by the hydrophilic, polar residue Threonine may change the protein property. Moreover, according to the Chou-Fasman parameters [15], [16], Alanine is one of the strongest helix formers whereas Threonine is one of the strongest β-sheet formers, which further supports the “probable” damaging effect of the amino acid change on the structure of protein. Furthermore, because the variant is located between the actin binding region and the ATP-binding (198–205^th^) domain of the myosin head-like domain of MYH14, the mutation is more likely to affect the binding activity of the protein. The identification of this additional novel mutation further confirms the crucial role of MYH14 in auditory function.

The *MYH14* gene was identified as a causal gene of DFNA4 deafness [17]. Until now, only 4 *MYH14* causative mutations have been reported in DFNA4 families [17]–[19]. In the present study, we identified a new mutation (c.541G>A; p.A181 T) in the *MYH14* gene. The phenotype of this family is different to that of the previously reported DFNA4 families. Affected members have a moderate to profound sensorineural hearing loss with a U-shaped audio profile. We have also identified a novel mutation (c.449C>T; p.A150 V) in the *WFS1* gene in another branch of the pedigree. Hearing loss in this family is nonsyndromic and segregated in an autosomal dominant manner. Hearing impairment is congenital and affected mid and high frequencies. The WFS1 (c.449C>T; p.A150 V) is carried by 1 out of 6489 exomes present on the Exome Variant server (MAF = 0.0077%). The wild-type nucleotide C at cDNA position 449 is also highly conserved, with a PhyloP score of 4.13, phasCons 0.927 and Polyphen-2 predicts this variant as ‘probably damaging’ with a score of 0.964. The protein product of WFS1, wolframin, is a membrane glycoprotein that is estimated to have nine helical transmembrane segments. It is primarily localized in the endoplasmic reticulum, with a hydrophilic extracytoplasmic N-terminus and a hydrophobic intracytoplasmic C-terminus [20]–[22]. Mutations in this gene are reported to be responsible for Wolfram Syndrome (WS), characterized by juvenile onset diabetes mellitus and optic atrophy, with 60% of the patients showing various degrees of hearing impairment by 20 years of age [23]. *WFS1* has also been linked to an autosomal dominant form of low-frequency sensorineural hearing loss (LFSNHL) [24]–[26], autosomal dominant optic atrophy (ADOA) [26]–[28], type 2 diabetes [29]–[31] and psychiatric problems [27], [32], [33]. Mutation analysis of *WFS1* in families with DFNA6/14 revealed that the identified pathogenic mutations in this disorder tend to be non-inactivating and cluster at the C-terminus or N-terminus of the protein domain [34], [35], whereas most of the pathogenic mutations in Wolfram syndrome patients are distributed over the entire coding region and are often inactivating mutations resulting in loss of function of the encoded protein [36]. The WFS1 (c.449C>T; p.A150 V) identified in the present study is located in the extracellular N-terminus domain of the wolframin protein. Three other mutations in the N-terminal cytoplasmic domain of the protein, p.R161Q, p.D171N and p.K193Q, have been associated with LFSNHL [34], [37] [45]. Other clinical features including optic atrophy have not been reported as concomitant features in families with LFSNHL. DFNA1, DFNA6/14/38 and DFNA54 are the loci so far reported as being associated with LFSNHL [38]. The progression of hearing impairment caused by a mutation in any of these three loci exhibits different characteristics. Patients carrying a mutation in the DFNA1 locus typically experience a rapidly deteriorating hearing loss progressing from low to involve all higher frequencies [39]. In contrast, those with a mutation in the DFNA54 or DFNA6/14/38 locus experience deafness propagating from low to high frequencies [40] or have no or mild progression beyond presbycusis [25], [41], [42]. The difference in phenotype across families may reflect influences of a modifier gene similar to DFNB1. The congenital, sharply sloping profound high frequency hearing loss in the individuals (IIC:1, IIIC:1, IIIC:2 and IIIC:3) carrying the WFS1 p.A150 V mutation is thus far unexplained, as we have ruled out involvement of *GJB2* and mitochondrial defect 12S rRNA in the phenotypic expression of the WFS1 p.A150 V mutation in those affected subjects. The fact that the carriers of the *WFS1* mutations associated with WS do not exhibit hearing loss and heterozygosity for other *WFS1* mutations resulting in nonsyndromic autosomal dominant LSFHNL reveals our incomplete knowledge of the function of the WFS1 protein and the impact of the different types of *WFS1* mutations. Another interesting point to be considered is a possible correlation between the location of the mutations within the WFS1 protein and the severity of the phenotype. Chaussenot et al (2011) [43] showed that the presence of at least 1 mutation that affects the carboxy-terminal tail of the protein seems to be more frequent in patients with neurologic symptoms whereas mutation in the N-terminal region was significantly correlated with the absence of neurologic complications. It has also been suggested that homozygosity or compound heterozygosity for missense mutation is more likely to lead to a “mild” phenotype whereas patients with one or more inactivating mutations would have a more severe phenotype. This distinction is rather arbitrary because only functional studies can determine which mutations are really inactivating. Our findings are broadening the mutational and phenotypic spectrum associated with the *WFS1* gene (Table 1).

Exome sequencing is not without limitations. For example, it does not detect mitochondrial disorders and may not capture each exon in every gene and insufficient depth of coverage in some regions can hinder detection of potential disease-causing genetic variants. The total proportion of gene variants that can be missed by current exome sequencing methods may be as high as 15%–20% [44].

In summary, we have combined both the Deafness Gene Mutation Detection Array and exome sequencing to identify deafness causative variants in this composite family with deaf by deaf mating. We first simultaneously screened the deaf patients for the 9 deafness common mutations using the allele-specific PCR based universal array, and then performed exome sequencing to identify the deafness causative gene in the mutation-negative cases. In the WES method, we consecutively filtered the variants by subjecting them to an analytical pipeline for high confidence variant calling and annotation. This simple filtering strategy rapidly led to the identification of causative variants in the *MYH14* and *WFS1* genes, and effectively demonstrates the potential of this combined approach to identify disease causative mutations in both novel and known genes in families with a small number of affected individuals.
